# Search for New Complex Sequences for the Implementation of an Aviation Group Interaction System of Small-Sized Airborne Radars

**DOI:** 10.3390/s26041245

**Published:** 2026-02-14

**Authors:** Vadim A. Nenashev, Renata I. Chembarisova, Aleksandr R. Bestugin, Vladimir P. Kuzmenko, Sergey A. Nenashev

**Affiliations:** Saint-Petersburg State University of Aerospace Instrumentation, 67, Bolshaya Morskaia Str., 190000 St. Petersburg, Russia; magna7746@gmail.com (R.I.C.); fresguap@mail.ru (A.R.B.); mr.konnny@gmail.com (V.P.K.); nenashev_sergey178@mail.ru (S.A.N.)

**Keywords:** aperiodic autocorrelation function, periodic autocorrelation function, sidelobes, phase modulation, complex M-sequence, compact on-board radar system, multi-position system, marked probing signal, aviation systems, cooperative probing

## Abstract

Recently, when forming radar video frames for surface mapping, group-interacting compact onboard radar systems (CORS) are increasingly being utilized. In this context, for the cooperative functioning of the group, each compact radar should use its own unique marked signal as the probing signal. This signal must be distinguishable in the common channel and should not destructively affect the probing signals emitted by other radars within the group. This organization allows for associating the marked signals reflected from the underlying surface with specific CORS in the group. This requirement arises because each compact onboard radar in the group emits a single probing signal and then receives all reflected signals from the surface emitted by the other CORS in the group. Such an organization of the group-based system of technical vision requires the search for and study of specialized marked code structures used for phase modulation of probing signals to identify them in the shared radar channel. The study focuses on the search for new complex M-sequences with lower sidelobe levels of the normalized autocorrelation function compared to traditional M-sequences. This is achieved by replacing the traditional alphabet of positive and negative ones with an asymmetric set consisting of complex numbers. Using numerical methods and computer simulations, the optimal complex values of the sequence with the minimum sidelobe level in the autocorrelation function are determined. In addition to correlation properties, the phase-modulated signals generated based on the new marked sequences are also investigated. The results obtained open new possibilities for the construction of a group-based technical vision system, enabling cooperative surface probing among all CORS in the interacting group.

## 1. Introduction

Currently, M-sequences are widely used in radar systems [1,2] to generate probing signals. These signals improve the accuracy of distance measurement to objects and ensure reliable detection by distinguishing the useful echo signal from noise. In communication systems, they serve to encode data to provide reliable synchronization between transmitting and receiving equipment. M-sequences also play an important role in the design of multi-position onboard control systems used in remote sensing aviation equipment [3,4,5,6,7,8,9,10,11].

High levels of side lobes (SL) in the autocorrelation function (ACF) can negatively affect the correct operation of CORS detection systems [12,13,14], leading to increased false alarms, reduced resolution, and decreased reliability of ground object identification. In this regard, an important task is the selection and formation of probing signals to ensure minimal SL in the ACF.

Various signal forms can be used to generate probing signals in CORS, such as linear frequency modulation, nonlinear frequency modulation, and phase-modulated (PM) signals [15]. This study focuses on PM signals, which enable the implementation of the principle of uniquely labeling probing pulses within a group of interacting CORS. Generating such signals requires code sequences with good correlation properties. In this regard, M-sequences represent a promising class of binary codes, characterized by high generation speed and the ability to create a large set of weakly correlated sequences, an important requirement for organizing the joint operation of several CORS.

The goal of this study is to search for new complex M-sequences where the SL levels of the normalized ACF (NACF) are lower than those of the traditional M-sequences, but with the same structure of positive and negative element positions. This reduction in side lobes is achieved by finding new complex values in the sequence structure through replacing the conventional symmetric alphabet of positive and negative ones with an asymmetric one.

To achieve the stated goal, the paper considers two cases of element substitution in the traditional sequence. In the first case, only the negative one is replaced with the sought-after complex number. In the second case, both the negative and positive ones in the M-sequence structure are replaced. In this study, it is necessary to analyze how the SL of the NACF changes and identify the complex values at which the side lobes of the NACF are minimized for the new complex M-sequences.

Numerical methods are employed to search for complex values of sequences for both cases. Computer experiments are conducted to evaluate the SL of the NACF. These experiments are based on the analysis of the expressions obtained that describe how each side lobe of the NACF changes as a function of the complex value assigned to the code element. Furthermore, phase modulation of the signal with the generated complex sequence is studied, and the correlation properties of the resulting signals are analyzed.

Section 2 examines existing analogues in the literature and analyzes their advantages and disadvantages. In Section 3, the aperiodic and periodic ACFs of traditional M-sequences are analyzed. The process for deriving expressions for the lobes is presented, from which graphs describing the sidelobe levels as a function of the introduced parameter are built for both the aperiodic and periodic ACFs in further research. In Section 4, dependencies of the side lobe levels of the periodic and aperiodic NACFs of the complex M-sequences are studied in relation to the new alphabet: {1; −exp(φi)} in the first case, and {exp(φ_1_i); −exp(φ_2_i)} in the second case. The values of the parameter φ vary in the range from 0° to 360°. Graphs illustrating the dependencies of the side lobes of the NACF on the parameter φ are provided. The minimal possible level of the side lobes of the NACF for the investigated M-sequences is identified. In Section 5, phase modulation of the probing signal with the generated complex M-sequences is carried out. Graphs of the NACF of the resulting signal are constructed. The minimum side lobe level of the NACF for the signal, corresponding to the minimum side lobe level of the complex M-sequence, is found, confirming proper phase modulation of the probing signal. In Section 6, the obtained results are discussed, further development recommendations are formulated, and areas for further application of the results in various fields are identified. In Section 7, the findings of the research are summarized, and the scientific and technical novelty of the results is highlighted.

## 2. Previous Works

In recent years, numerous algorithms for searching and generating sequences and signals modulated by them have been developed for radar and telecommunications systems, aimed at improving their correlation characteristics. The type of sequence and the corresponding signals affect the range resolution, noise immunity, and the ability to separate these signals in multi-position radar systems and multi-user communication systems.

Among the best-known classes of sequences with good autocorrelation properties are Barker codes, which have low sidelobe levels for their length [16,17,18,19]. However, their use is limited by the maximum known length (13 elements). This makes them unsuitable for modern wideband detection systems.

Polyphase sequences, such as the Frank [20,21] and Chu [22,23,24,25] codes, exhibit low sidelobe levels in their autocorrelation functions and are used in phase-shift keying systems. Despite their strong correlation properties, these sequences often require complex hardware implementation and do not always meet the requirements for shared use in group operations in multi-position systems.

To create more universal solutions, algorithms were developed that generate codes of any length with the required correlation characteristics. One such algorithm is the cyclic algorithm, which minimizes sidelobes in the frequency domain [26]. Although it has proven effective, it does not work with the original target function but rather with its approximation; hence, the level of sidelobe suppression can be significantly worse than the theoretically achievable limit, which is not always acceptable.

To address this drawback, a class of algorithms based on minimizing sidelobe levels was proposed [27]. Although these algorithms operate directly on the objective functional, they exhibit slow convergence, which complicates their use for generating long sequences or sets of sequences in real time.

Although these algorithms have certain advantages, their practical application poses several difficulties. Some algorithms can account for weighted correlation shifts [14] or handle a limited number of distinct elements in the sequence alphabet [16,17,18,19]. Other algorithms [26] operate not with an exact, but with an approximate objective function. All this complicates the minimization of sidelobe levels during signal compression and the implementation of probing in a multi-position mode.

Similarly, genetic algorithms, a class of heuristic optimization methods, mimic the principles of natural selection to navigate through a large parameter space in search of optimal waveform weights, thereby minimizing sidelobe peaks [28]. However, such methods often require a huge amount of computation and do not guarantee convergence to an optimal solution, especially for sequences of large length and dimension.

The algorithm proposed in this study distinguishes itself from other similar algorithms in its conceptual basis. Instead of complex iterative optimization of the entire sequence or working with approximated functionals, it is based on modifying the alphabets of existing and well-studied sequences. This provides several key advantages.

First, the algorithm initially takes into account the structure of both the aperiodic and periodic ACFs (AACF and PACF). The search for optimal complex parameters is based on analytical expressions for the SL of both correlation functions, which allows us to find solutions that simultaneously improve their characteristics, as shown in Section 4 and Section 5.

**Figure 1:**
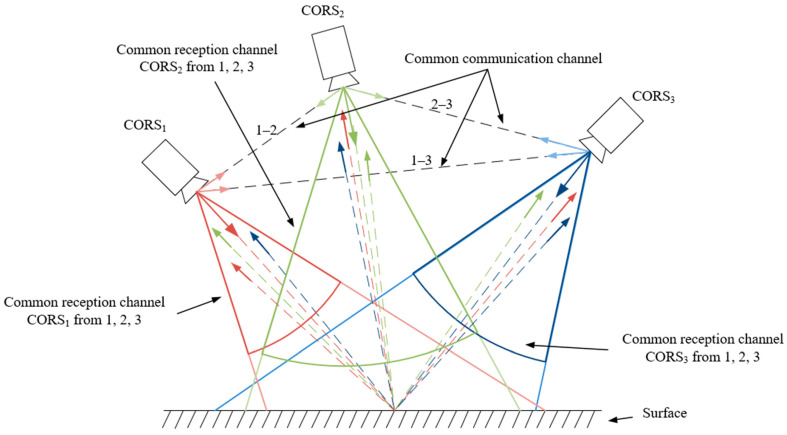
Layout of several CORS in space.

Secondly, in its generalized form, the algorithm can be extended to jointly optimize not only the autocorrelation but also the cross-correlation properties of a set of sequences. This is achieved by introducing additional complex parameters for each sequence in the group and formulating an objective function that minimizes the maximum SL level of both autocorrelation and cross-correlation. Thus, the proposed algorithm is positioned as a convenient tool for searching codes and generating signal-code structures based on them for the implementation of a multi-position system consisting of small-sized airborne radars interacting with each other (Figure 1). Signals from different airborne radars are highlighted in different colours.

Thirdly, searching by one, two, or more parameters for a fixed M-sequence structure is a relatively low-cost procedure, which opens up prospects for its application in systems requiring the generation of large families of codes.

Thus, an analysis of existing approaches highlights the scientific novelty and practical value of the algorithm for searching for new complex sequences. The key distinction of the proposed algorithm lies in its ability to selectively and simultaneously reduce the sidelobe levels of both the periodic and aperiodic autocorrelation functions through parametric modification of the code alphabet, as well as its potential for generalization to the optimization of cross-correlation properties across a group of signals. Taking these factors into account allows us to significantly reduce the levels of autocorrelation side lobes and cross-correlation. Consequently, our approach represents a significant new alternative for applications where consideration of the above factors is paramount. This makes this search algorithm promising, primarily for use in modern multi-position and communication systems that impose strict requirements on signal separation with their binding to a specific emitting position and to the lobe level after compression. Since optimizing the sidelobe level of compressed radar signals remains a complex and pressing problem, this study builds on existing research and aims to further explore the potential for new sequences and encoded radar and telecommunication signals that meet modern requirements.

## 3. Autocorrelation Function of the M-Sequences

**Figure 2:**
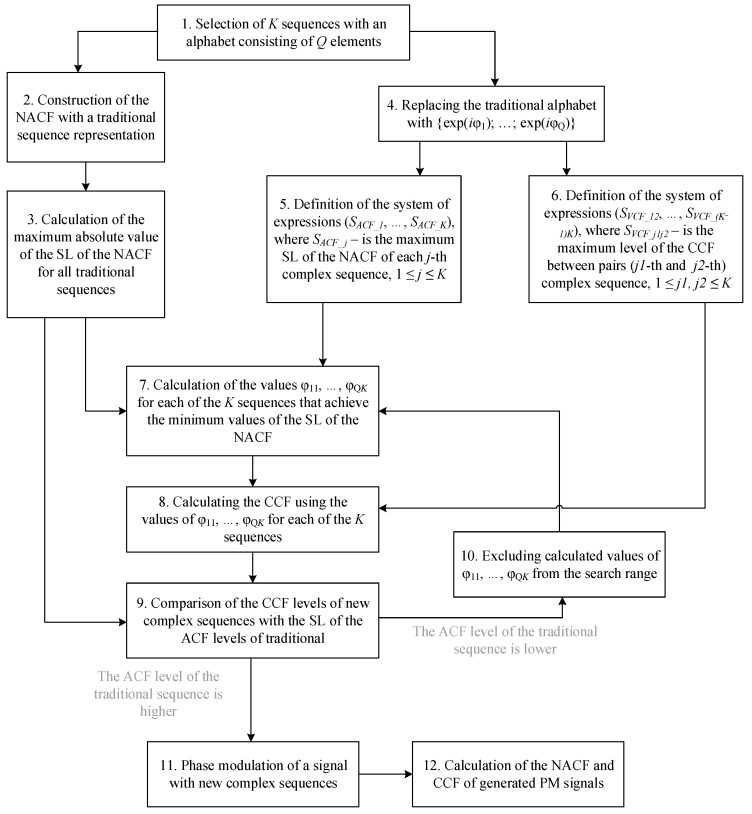
Schematic diagram of the general algorithm for modifying the sequence alphabet.

In general, the developed algorithm can operate with multiple sequences generated using an arbitrary alphabet containing any number of elements (two-position, three-position, etc.). The algorithm is designed to modify a traditional sequence, such that, with a modified alphabet, its ACF has improved characteristics compared to the original version, namely, a lower level of SL. Furthermore, when working with multiple modified sequences of the same length, the algorithm reduces the cross-correlation function (CCF) levels between them to values below the ACF’s maximum sidelobe. This improves noise immunity and separability of modulated signals in modes of joint irradiation by several CORS combined into an interacting group. The general structure of the new sequence search algorithm is shown in Figure 2.

**Figure 3:**
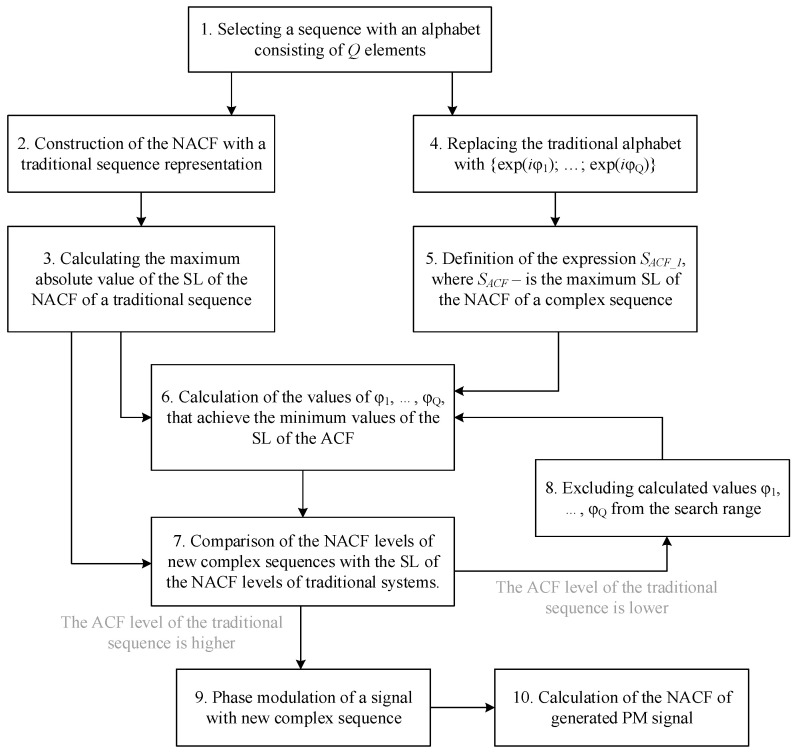
Schematic diagram of the sequence alphabet modification algorithm.

The study further discusses a specific version of the algorithm, which focuses on improving the ACF characteristics of the modified sequence. The structure of the specific algorithm, which is further discussed in this study for M-sequences, is shown in Figure 3.

When implementing a multi-position system of interacting CORS, various signal-code constructs are often used to generate probing and synchronizing signals with the required correlation characteristics. Both binary and polyphase codes are used in onboard systems [15]. Binary codes include, for example, Barker, Gold, and M-sequence codes. Polyphase sequences of Frank and Chu, which have low levels of SL in the ACF, are widely used as phase codes. For CORS, the simplicity of hardware implementation and the stability of characteristics during group operation of interconnected CORS are of particular importance.

Therefore, preference is often given to M-sequences, which, despite their long length, provide low levels of SL in the ACF, uniform energy distribution across the spectrum, and the possibility of flexible parameterization without significantly complicating the transmitter and receiver equipment [29,30,31,32,33]. For M-sequences, the maximum value of the ACF occurs only at a zero shift, while for any other shifts, the correlation approaches zero.

It should be noted that for constructing a multi-position CORS system, code-modulated signals are used, both when selecting the probing signal and when selecting the synchronizing signal used in the frame preamble, which is necessary for organizing communication channels between the group of CORS, as well as for their management [34,35,36,37,38,39,40].

Therefore, both the properties of the AACF and PACF need to be studied. The AACF should be analyzed for the implementation of Earth’s surface probing modes, including group modes that use modulated signals. The PACF should be studied when organizing communication channels, i.e., when combining several CORS into an interacting group.

The ACF of the sequence x(n) is denoted as R(k) and is computed using expression (1) for the PACF of sequences, and expression (2) for the AACF.

For the PACF calculation:

R_p_(k) = ∑x(n) × x(n + k), (1)

where n varies from 0 to N − 1 (N—sequence length), and x(n + k) is the sequence element shifted by k positions. If the sequence element with index n + k goes beyond the sequence length, periodic continuation of the sequence is used.

For the calculation of the AACF:

R_a_(k) = ∑x(n) * x(n + k), (2)

where k varies from 0 to N − 1, and n varies from 0 to N − k − 1. In this case, the summation is performed only over those n indices for which n + k remains within the sequence length N.

In general form, the process of obtaining expressions describing the sidelobes of the AACF and PACF is presented in Table 1 and Table 2, respectively. This example is presented for an M-sequence of length N = 4. The number of sidelobes of the ACF can be calculated using Equation (3):

(N − 1)/2(3)

For convenience, each sidelobe of the ACF is assigned an index m, with the main lobe having index 0, and the SL having indices −3, −2, −1, 1, 2, 3. As seen from Table 1 and Table 2, there is a relationship between the analytical expressions for the main lobe and the SL of the ACF, which can be expressed using Equation (4):

r_p_(m) = r(m) + r(m − N), (4)

where m is the index of the autocorrelation function lobe, rp(m) is the value of the m-th SL of the PACF, r(m) is the value of the m-th SL of the AACF, and N is the sequence length.

In this context, the CORS hardware is subject to specific requirements regarding the choice of probing signal, which directly influence the levels of the SL of the AACF [41,42,43]. For Earth’s surface-probing modes, AACF analysis is of paramount importance. Its key advantage is a well-defined main lobe at zero shift combined with the minimal SL levels, which ensures high resolution in the range coordinate.

For ensuring interference-resistant data exchange in wireless communication channels between CORS positions, the PACF should be analyzed. The PACF analysis will improve the survivability and fault tolerance of the data exchange system within the interacting group of CORS [44].

Thus, the analysis of the AACF properties is necessary for implementing group probing signal modes, while the corresponding analysis of the PACF is necessary for implementing communication channels. This will allow the creation of multi-position radar systems optimally combining location and information exchange functions within the interacting group. Such a combination will expand the functional capabilities of individual CORS units, and therefore, the search for new signal-code constructions, both for probing and data exchange, is a modern and relevant task.

## 4. Search for New Complex M-Sequences

When studying M-sequences, it is necessary to minimize the level of SL of the NACF. This can be achieved by searching for new values in its code structure. This study proposes replacing the elements of the existing traditional symmetric alphabet {1; −1} in the M-sequence structure with an asymmetric alphabet that can consist of both real and complex values. In complex form, the exponential form of the code elements is used in the form exp(φi), where φ is the angle of the unit vector on the complex plane. φ can also be used as the initial phase for phase modulation of elementary pulses when forming a signal-code structure [45,46,47,48,49,50,51].

Next, traditional M-sequences of length N = 7, 15, 31, 63, 127, 255, 511 are examined as an example illustrating the process of modifying code values to a complex version, using four M-sequences (two M-sequences of length 7 and 15). Table 3 lists the generator polynomials [52] and the traditional sequences generated from them with a symmetric alphabet consisting of −1 and +1.

In [53], optimal replacements of negative ones with the real element “a” in the structures of M-sequences of length N = 15, 31, 63, 127, 255, 511 were determined. Below, two options for changing the traditional alphabet {1; −1} of M-sequences to: {1; −exp(φi)} and {exp(φ1i); −exp(φ2i)}, respectively, will be considered.

### 4.1. Replacing One Negative Element in the Alphabet of Traditional M-Sequences with a Complex Value

In the first variant, the negative 1 in the code structure is replaced with a complex value—«−exp(φi)», while the positive 1 remains in its place without changing its value. An example of such a replacement of the alphabet of sequences from Table 3 is presented in Table 4.

**Figure 4:**
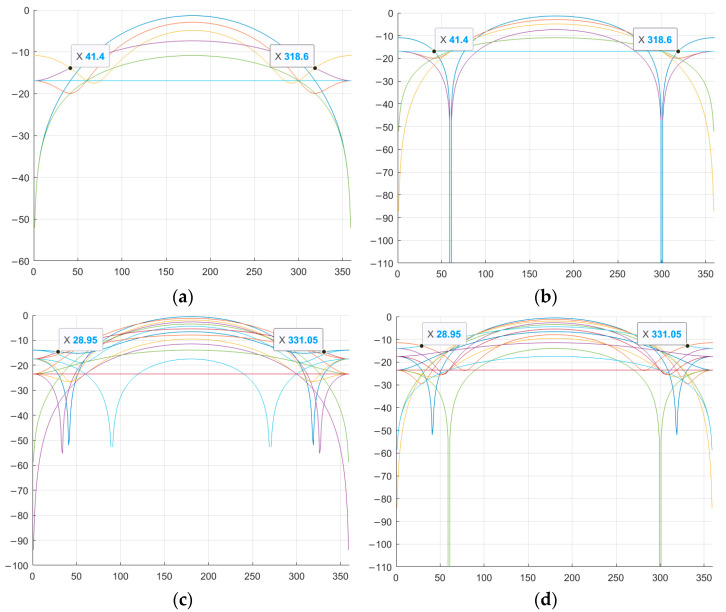
Dependence of the aperiodic NACF of a complex M-sequence on the parameter φ: (**a**) M-sequence №1; (**b**) M-sequence №2; (**c**) M-sequence №3; (**d**) M-sequence №4.

Next, it is necessary to find the optimal value of φ at which the SL of the NACF level is lowest. That is, to search for and determine the specific φ value from the 0–360° range at which the SL will have the minimum level. Graphical representation of the SL levels of the aperiodic NACF of M-sequences from Table 4 with the alphabet {1; −exp(φi)} is shown in Figure 4. The horizontal axis shows the value of φ from the range 0–360°, the vertical axis shows the value of SL of the NACF in decibels. Figure 4 shows the values of all sidelobe levels of the aperiodic NACF for each value of φ in a given range. To determine the optimal parameter φ, the maximum sidelobe levels for each φ are analyzed, after which the φ value is selected at which this largest sidelobe takes on the minimum possible value.

**Figure 5:**
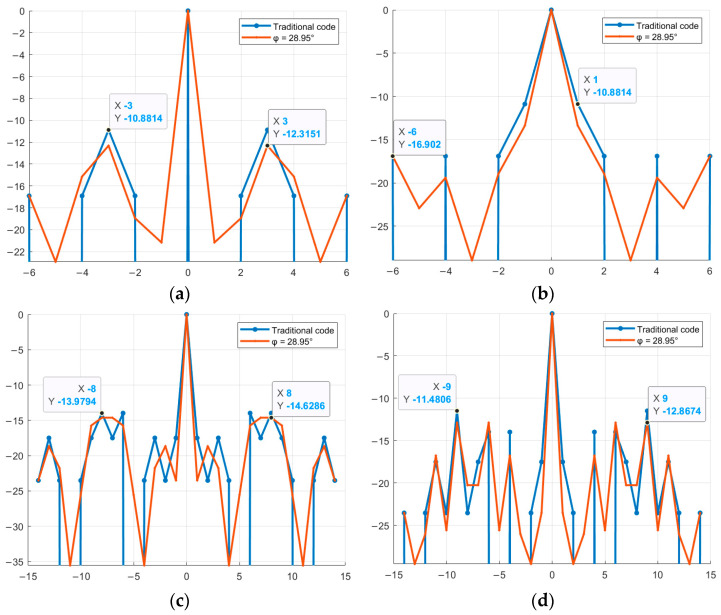
Comparison of the level of aperiodic NACF of the traditional M-sequence and its complex version depending on the parameter φ: (**a**) M-sequence №1; (**b**) M-sequence №2; (**c**) M-sequence №3; (**d**) M-sequence №4.

As can be seen from Figure 4, for M-sequences of the same length, the optimal values of φ are the same initial phases: φ_1_ = 41.4° and φ_2_ = 318.6° for N = 7, φ_1_ = 28.95° and φ_2_ = 331.05° for N = 15. Figure 5 shows a comparison of the aperiodic NACF with traditional M-sequences. The horizontal axis shows the indices of the m petals, and the vertical axis shows the values of the petal levels of the NACF of the sequences in decibels.

**Figure 6:**
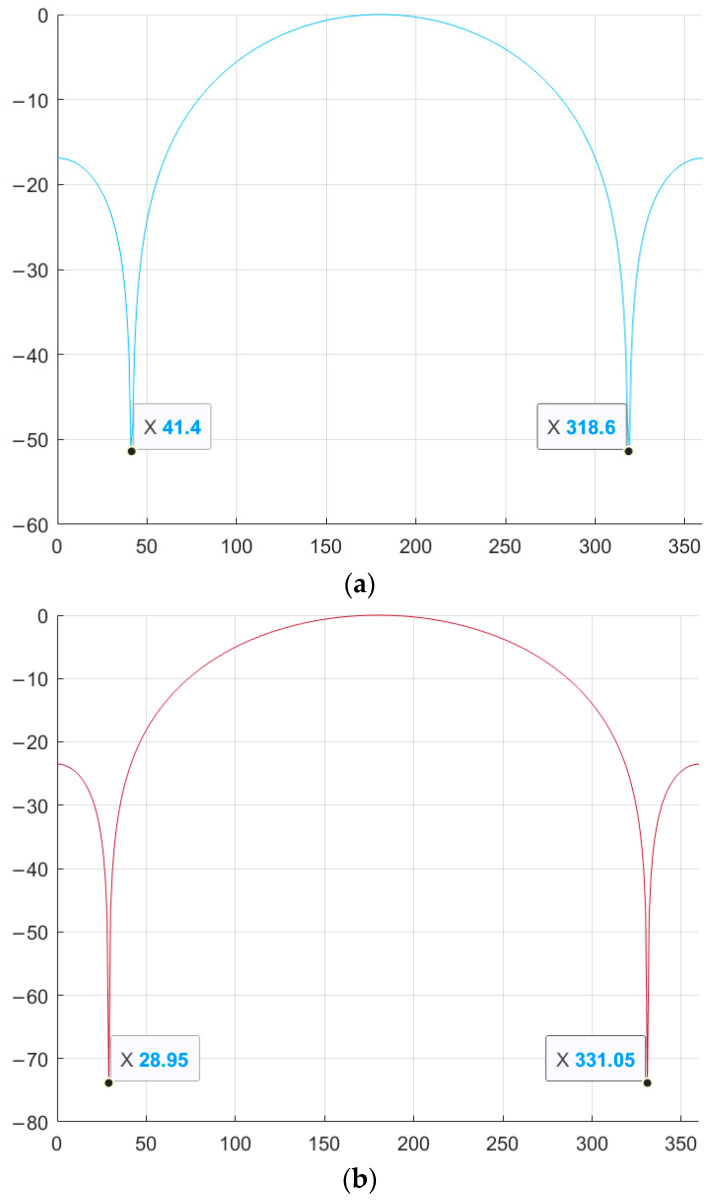
Dependence of the periodic NACF of a complex M-sequence on the parameter φ: (**a**) M-sequence of length N = 7; (**b**) M-sequence of length N = 15.

As shown in Figure 5, certain values of the parameter φ allow reducing the SL of the NACF of traditional M-sequences. Thus, for a traditional M-sequence of length 7, the level of the SL of the NACF is −10.88, and for the obtained complex M-sequences, the level of the SL of the NACF is −13.89 and −16.9 for M-sequences №1 and №2, respectively. Therefore, the difference between the values is 3.01 and 6.02 dB. For M-sequences of length 15, the difference between the values is 0.65 and 1.39 for M-sequences №3 and №4, respectively. Similar to the studies above for the aperiodic NACF, Figure 6 shows the change in the SL of the periodic M-sequence NACF. Similar to Figure 4, the horizontal axis of Figure 6 displays the value of φthe vertical axis displays the SL of the NACF in decibels.

**Figure 7:**
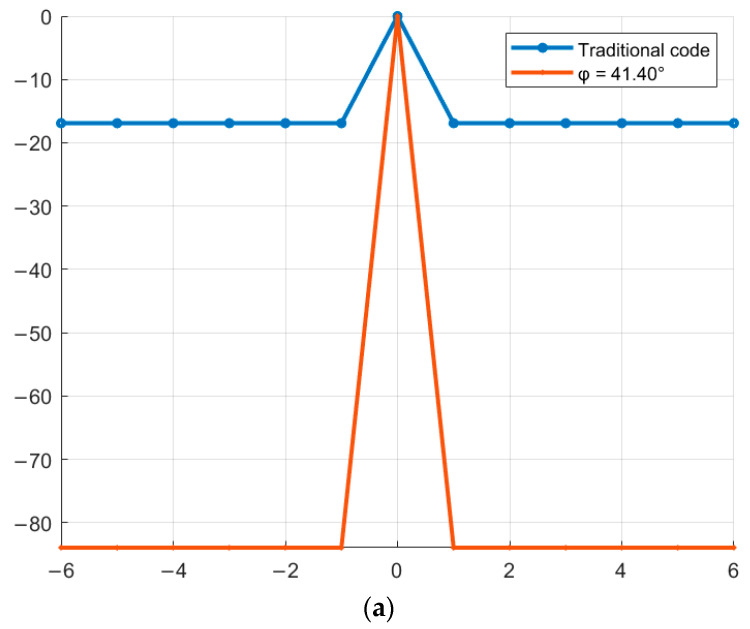

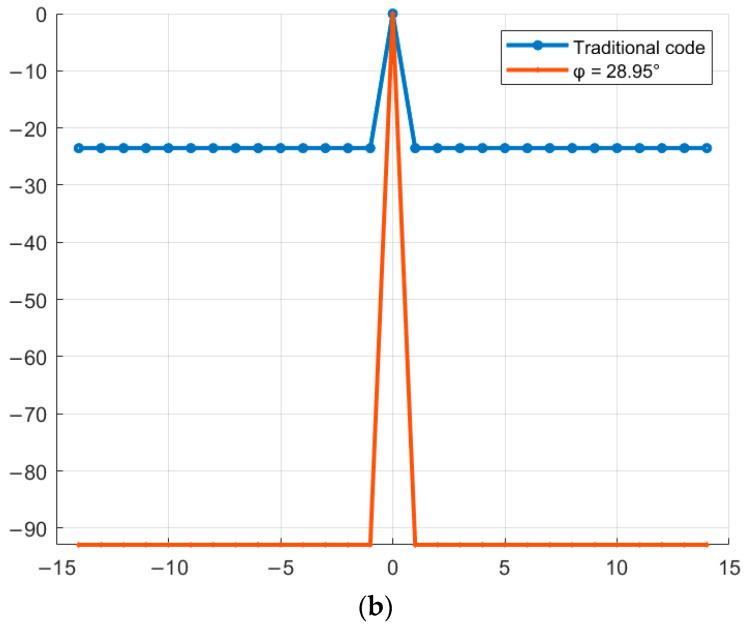
Comparison of the level of the periodic NACF of the traditional M-sequence and its complex version depending on the parameter φ: (**a**) M-sequence of length N = 7; (**b**) M-sequence of length N = 15.

As shown in Figure 6, for the periodic NACF, the optimal values are φ_1_ = 41.4° and φ_2_ = 318.6° for the M-sequence with length N = 7 and φ_1_ = 28.95° and φ_2_ = 331.05° for the M-sequence with length N = 15. Thus, the optimal values for the periodic NACF coincide with the optimal values for the aperiodic NACF. Figure 7 shows a comparison of the BL level of the periodic NACF of the traditional and complex M-sequences. The horizontal axis displays the φ value from 0 to 360°, and the vertical axis displays the SL of the NACF value in decibels.

As can be seen from Figure 7, complex M-sequences can significantly reduce the level of SL of the NACF. For the traditional M-sequence of length N = 7, the SL of the NACF level is −16.90, and for the obtained complex M-sequences, the SL of the NACF level is −112.04. Therefore, the difference between the values is 95.14. For M-sequences of length N = 15, the difference between the values is 69.77 (the SL of the NACF level is −23.52 for the traditional sequence and −93.29 for the complex one).

### 4.2. Replacing Two Elements in the Alphabet of Traditional M-Sequences with Complex Values

The second variant of alphabet replacement for M-sequences replaces the positive 1 with «−exp(φ_1_i)» and the negative 1 with «exp(φ_2_i)». An example of replacing the alphabet of the previously discussed sequences is presented in Table 5.

**Figure 8:**
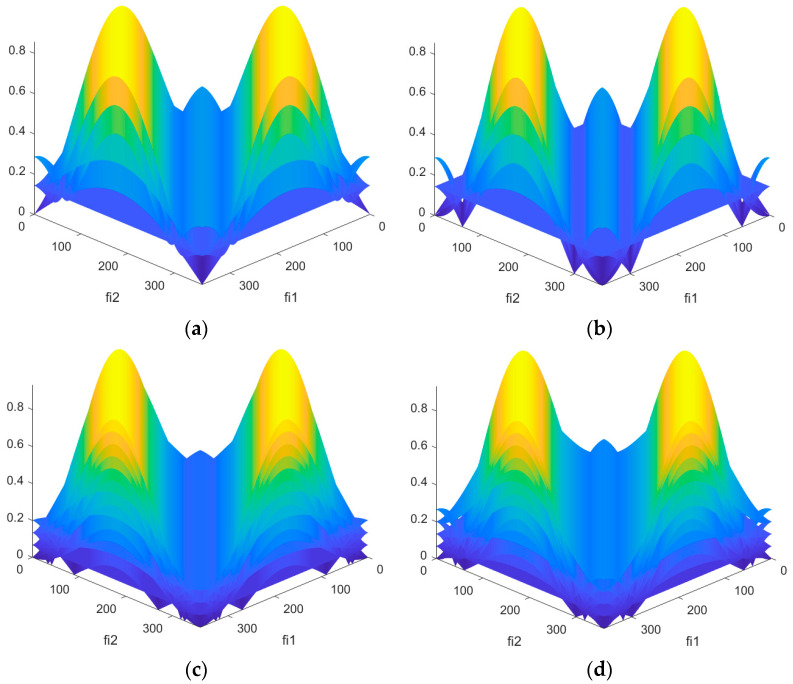
Dependence of the aperiodic NACF of a complex M-sequence on the parameters φ_1_ and φ_2_: (**a**) M-sequence №1; (**b**) M-sequence №2; (**c**) M-sequence №3; (**d**) M-sequence №4.

Similar to the variant with the replacement of one alphabet element, it is necessary to determine the optimal values of the pair {φ_1_, φ_2_} from the range 0–360°, at which the SL of the NACF level is the lowest. A graphical representation of the SL of the NACF level for M-sequences is shown in Figure 8. The vertical axis shows the SL values of the NACF for φ_1_ and φ_2_.

**Figure 9:**
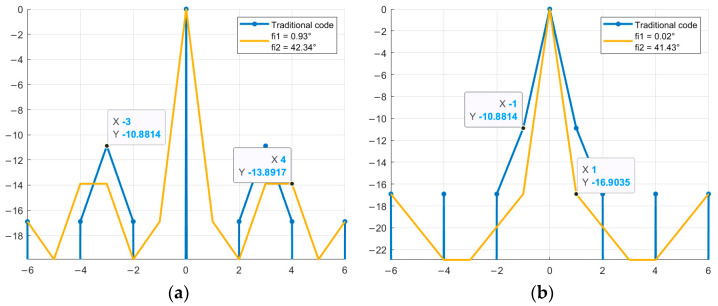

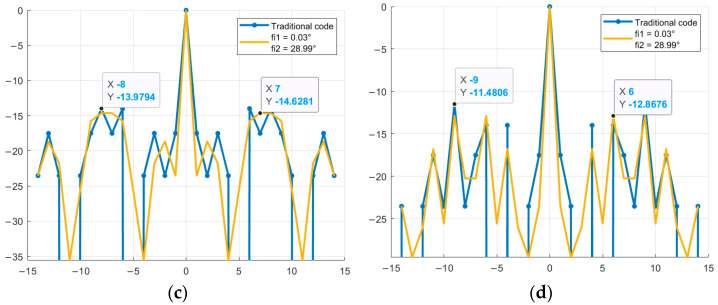
Comparison of the level of aperiodic NACF of the traditional M-sequence and its complex version depending on the parameters φ_1_ and φ_2_: (**a**) M-sequence №1; (**b**) M-sequence №2; (**c**) M-sequence №3; (**d**) M-sequence №4.

When replacing two elements from the M-sequence alphabet, the optimal values of φ_1_ and φ_2_ are significantly greater than when replacing a single element. Thus, for M-sequences of length N = 7, the number of pairs {φ_1_, φ_2_} exceeds 700. An important pattern is observed: for all the resulting optimal pairs {φ_1_, φ_2_}, the difference between their values is, on average, 42, i.e., |φ_1_ − φ_2_| ≈ 42. To check the reduction in the SL of the NACF, the first found pairs {φ_1_, φ_2_} are used (Figure 9). The horizontal axis displays the φ value from 0 to 360°, and the vertical axis displays the SL of the NACF value in decibels.

**Figure 10:**
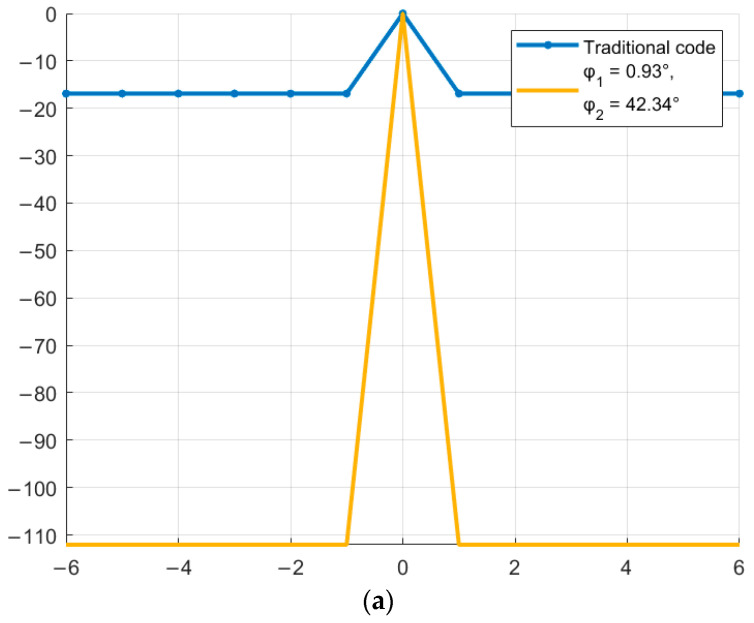

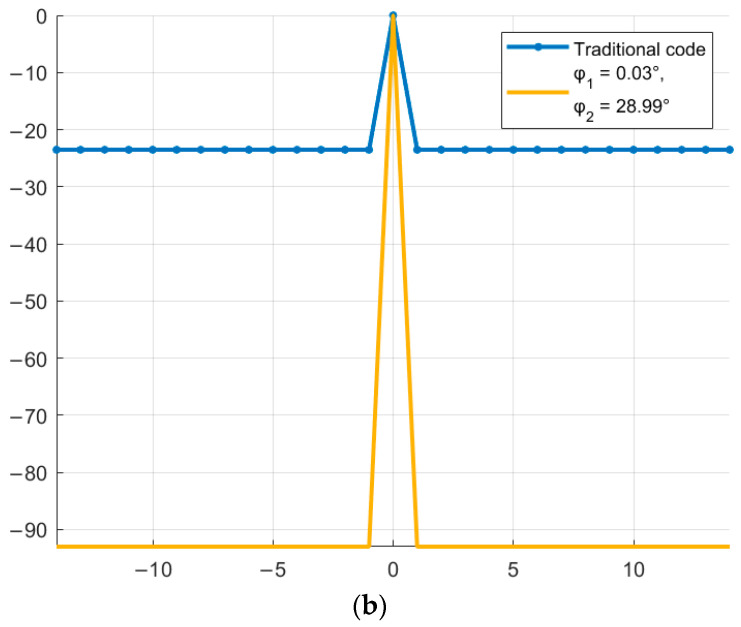
Comparison of the level of the periodic NACF of the traditional M-sequence and its complex version depending on the parameters φ_1_ and φ_2_: (**a**) M-sequence of length N = 7; (**b**) M-sequence of length N = 15.

Figure 5 and Figure 9 are similar. However, Figure 5 shows the change in the SL levels of the aperiodic NACF as a function of one parameter, φ, while Figure 9 shows the dependence on two parameters, φ1 and φ2. This suggests that the lowest SL values of the NACF can be achieved by using either one or two parameters, φ. Figure 10 shows a comparison of the SL of the periodic NACF of a traditional and complex M-sequence, depending on two parameters. The horizontal axis displays the φ value from 0 to 360°, and vertical axis displays the SL of the NACF value in decibels.

As can be seen from the study, the reduction in the SL of the NACF of M-sequences when replacing either one or two alphabet elements occurs by approximately the same values, which is also confirmed by the experimental results presented in Figure 5, Figure 6, Figure 7, Figure 8, Figure 9 and Figure 10. Below is the average reduction in the SL level for M-sequences of length N = 7, 15, 31, 63, 127, 255, 511. Table 6 presents the studied M-sequences, and Table 7 presents the reduction in the SL level of the NACF for all sequences.

An analysis of the numerical results in Table 7 shows that using certain complex M-sequences, it was possible to reduce the SL level of the normalized aperiodic and periodic ACF compared to the same level obtained using traditional representations of this code.

Therefore, for example, the greatest decrease in the maximum SIDE LOBE for the normalized AACF was 6.01 dB for the M-sequence of length N = 7 with the generating polynomial *x*^3^ + *x* + 1, while for the normalized PACF it was 95.14 dB for the M-sequence of length N = 7. With an increase in the length of the M-sequence, the SL level of the NACF decreases, which will increase the probability of correct signal detection for a given probability of false alarm, that is, against the background of internal noise of the receiving device.

Based on this, it should be concluded that it is advisable to use new complex element values to modify M-sequences.

## 5. Phase Modulation of a Signal by Complex M-Sequences

In modern CORS, one of the main requirements is to increase the range resolution. For this purpose, complex broad-spectrum probing signals are used in practice, which improves the accuracy and reliability of range estimation to ground targets [54,55,56,57,58,59,60,61,62,63].

The choice of the signal modulation type is also important [64], which affects the range resolution when detecting ground objects.

Next, the process of generating and compressing new signal-code constructs should be considered. Afterwards, the maximum sidelobe levels of normalized compressed signals modulated in phase by traditional and new complex M-sequences should be compared. The previously obtained values of the initial phase φ for the new complex sequences make it possible to generate phase-modulated signals with lower levels of SL of the NACF compared to traditional ones. Compression of the modulated signals under consideration is carried out using traditional processing. That is, based on matched filtering based on the maximum level of SL.

**Figure 11:**
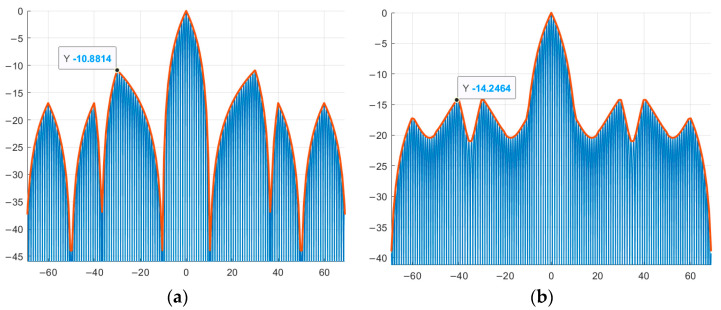

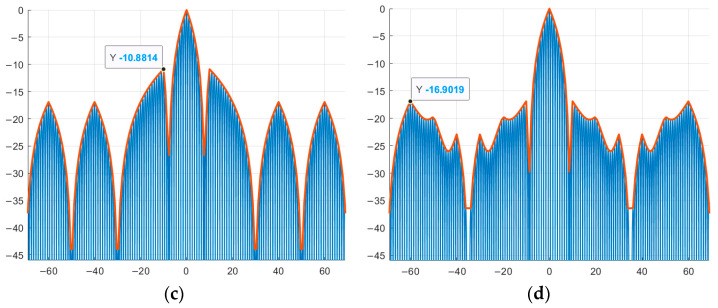
Phase modulation of a signal by aperiodic traditional and complex M-sequence: (**a**) traditional M-sequence №1; (**b**) complex M-sequence №1; (**c**) traditional M-sequence №2; (**d**) complex M-sequence №2.

As an example, complex M-sequences of length N = 7 with the alphabet {1; −exp(φi)} (Figure 11). The compressed phase-modulated signal is shown in blue, and the envelope of the high-frequency content of the compressed signal, phase-modulated by new complex sequences, is shown in red. The point corresponding to the maximum value of the SL of the compressed signal is marked on the graph. The vertical axis shows the values of the SL of the compressed signals, and the horizontal axis shows the signal duration.

**Figure 12:**
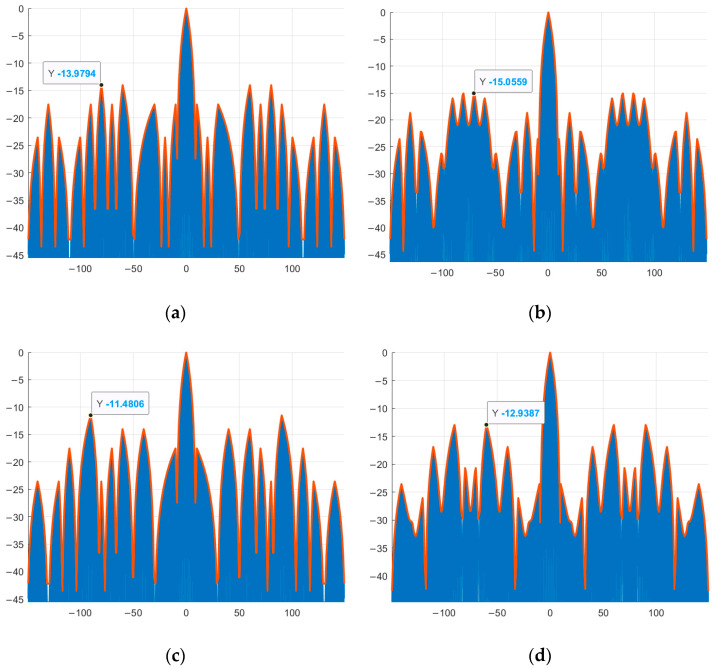
Phase modulation of a signal by aperiodic traditional and complex M-sequence: (**a**) traditional M-sequence №3; (**b**) complex M-sequence №3; (**c**) traditional M-sequence №4; (**d**) complex M-sequence №4.

Similar to Figure 11, complex M-sequences of length N = 15 with the alphabet {1; −exp(φi)} are considered below (Figure 12). The vertical axis shows the values of the SL of the compressed signals, and the horizontal axis shows the signal duration. The compressed phase-modulated signal is shown in blue, and the envelope of the high-frequency filling of the compressed signal, modulated in phase by new complex sequences, is shown in red. The point corresponding to the maximum value of the SL of the compressed signal is marked on the graph.

Comparing Figure 5a with Figure 11a,b and Figure 5b with Figure 11c,d and Figure 5c with Figure 12a,b and Figure 5d with Figure 12c,d, it is evident that the maximum SL level of the aperiodic NACF of the complex M-sequence and the phase-modulated signal based on it coincide within the computational error, which indicates that they correspond to the theoretical data presented in Table 7.

Further, Table 8 presents the numerical results of the SL levels of compressed signals modulated in phase based on traditional and new complex M-sequences of length N = 7, 15, 31, 63, 127, 255, 511, as well as the level of reduction compared to the latter.

From the analysis of the numerical results in Table 8, it follows that when using the developed scheme to search for new complex values within the structure of M-sequences (see Figure 1), it is possible to reduce the SL levels of compressed signals compared to traditional M-sequences. For example, the greatest reduction in the maximum SL of the aperiodic NACF was 6.01 dB for the M-sequence of length N = 7 with the generating polynomial *x*^3^ + *x* + 1, and the least reduction in the SL was 0.04 dB for the polynomial *x*^7^ + *x* + 1 generating the M-sequence of length N = 127. For the normalized PACF, the greatest reduction in SL was 95.10 dB for the M-sequence of length N = 7, and the least reduction was 54.54 dB for the M-sequence of length N = 255. The remaining similar numerical results lie between the specified SL levels for the AACF and PACF, respectively.

Moreover, the compressed signal shape is preferable for signals phase-modulated by the new complex M-sequence compared to traditional signals due to the absence of sharp slopes at the BL boundaries. This absence allows the envelope of the compressed signal to be constructed more quickly than for signals with slopes.

Based on this, is advisable to use the developed algorithm to search for new values within the structure of traditional M-sequences. The results of this study demonstrate the broad possibilities arising from the asymmetry of element values within the structure of the original traditional sequence.

Thus, the problem of generating new codes for modulating probing signals with the desired autocorrelation parameters has been solved. This opens the possibility of constructing display modes based on complex data processing in group interaction systems.

## 6. Discussion

The algorithm described in this paper has successfully demonstrated its effectiveness on M-sequences, suggesting its applicability to codes with an extended alphabet. Generally, it can be used to search for new element values in sequences over arbitrary alphabets, and is not limited to binary codes. Section 2 presented the general concept of searching for new sequences with an arbitrary number of alphabet elements; however, M-sequences were chosen specifically for testing the algorithm in this paper, as they are among the most common in detection systems.

The proposed algorithm reduces the sidelobe level of the autocorrelation function while preserving the key advantages of M-sequences: ease of generation, a large family of weakly correlated codes, and structural predictability, which are important for constructing group systems of interacting CORS. The results confirm that the generated complex M-sequences exhibit significantly lower sidelobe levels than traditional binary representations, including in the phase-modulation mode of probing signals, thereby improving the resolution and noise immunity of multi-position radar systems.

In addition to low SL of the periodic and aperiodic ACF of complex M-sequences, it is also possible to achieve a fairly low level of the CCF of complex M-sequences. In general, the algorithm is designed to simultaneously improve both the ACF and the CCF when working with multiple sequences, which is especially important for group and multi-channel modes.

In this regard, future research aims to find values of φ that ensure not only low sidelobe levels in the normalized autocorrelation function, but also the required low cross-correlation lobes between several M-sequences of the same length, formed by different generator polynomials. A key requirement is maintaining a balance among these parameters to enable the use of multiple signals in a single radar channel.

For example, comparisons of compression results for several types of probing signals are known. Signals with frequency modulation (linear and nonlinear) and signals with phase modulation were compared. Traditional M-sequences (e.g., of length 63) were used as an example of phase-shift key signals. The signal durations were identical. Analysis showed a gain in the “maximum sidelobe level” parameter. The difference between compressed linearly frequency-modulated signals and signals based on the M-sequence was 2.15 dB. The difference between nonlinearly frequency-modulated signals and the traditional M-sequence reached 1.91 dB [65].

Moreover, this work demonstrated that the SL level of the compressed signal modulated by the new complex M-sequence is lower than the SL level of the compressed signal modulated by the traditional M-sequence. This suggests that the performance of compressed signals modulated with new complex M-sequences may be better for linearly and nonlinearly frequency-modulated signals and for other lengths.

A promising direction is to expand the algorithm to jointly optimize multiple modulation parameters, such as phase, frequency, and amplitude, while simultaneously monitoring the SL of the ACF and CCF. In this case, the CCF lobe levels should be below the ACF’s maximum SL, which helps ensure high separability of modulated signals in multi-position joint sensing modes.

The approach in this paper for finding new values for M-sequence constructs can also be used as a direction for further research. This approach can be applied to finding new values for other types of sequences, such as pseudorandom sequences, both binary and with larger alphabets.

In addition to communications, radar, and radio navigation, these signals can be used to implement group interactions between land-, sea-, and underwater-based swarms. Using a new approach to searching for complex sequences with improved correlation properties reduces the mutual interference between signals in a single channel and increases the resilience of group systems to destructive interference. Further research can aim to modify the code structure to ensure both a low SL for the NACF and low mutual interference in a single channel.

## 7. Conclusions

The study resulted in an assessment of the properties of the autocorrelation functions of complex M-sequences, including an analysis of their periodic and aperiodic components. An approach based on replacing the traditional sequence alphabet with an asymmetric complex alphabet was developed, significantly reducing sidelobe levels in the autocorrelation function.

It is shown that the proposed algorithm is generally applicable to finding new sequences with an arbitrary number of elements in its alphabet. Furthermore, this algorithm can be used not only for binary codes, but also for sequences with an extended set of values.

The practical significance of the obtained results lies in their application to improving the quality of airborne radar and communication systems, where minimizing the level of SL of the NACF is a key requirement. The newly discovered complex M-sequences demonstrated high efficiency in computer experiments, further enabling the generation of signal-code structures for group systems used in airborne monitoring of Earth’s surface.

Thus, the use of complex M-sequences and signals modulated by them confirmed the improvement of their correlation properties by searching for new values in the structure of the M-sequence by replacing the traditional alphabet {1; −1} with an asymmetric complex one.

The obtained results provide a basis for further expanding the algorithm to multi-position modes, with simultaneous optimization of the SL for the ACF and CCF levels. This will improve noise immunity, signal separability, and the operational efficiency of multi-position radar systems combined into an interacting array.

## Figures and Tables

**Table 1:** Process of obtaining expressions for the sidelobes of the AACF.

	a_1_	a_2_	a_3_	a_4_			
m	−3	−2	−1	0	1	2	3
a_4_*	a_1_ × a_4_*	a_2_ × a_4_*	a_3_ × a_4_*	a_4_ × a_4_*			
a_3_*		a_1_ × a_3_*	a_2_ × a_3_*	a_3_ × a_3_*	a_4_ × a_3_*		
a_2_*			a_1_ × a_2_*	a_2_ × a_2_*	a_3_ × a_2_*	a_4_ × a_2_*	
a_1_*				a_1_ × a_1_*	a_2_ × a_1_*	a_3_ × a_1_*	a_4_ × a_1_*
	a_1_ × a_4_*	a_2_ × a_4_* + a_1_ × a_3_*	a_3_ × a_4_* + a_2_ × a_3_* + a_1_ × a_2_*	a_4_ × a_4_* + a_3_ × a_3_* + a_2_ × a_2_* + a_1_ × a_1_*	a_4_ × a_3_* + a_3_ × a_2_* + a_2_ × a_1_*	a_4_ × a_2_* + a_3_ × a_1_*	a_4_ × a_1_*

**Table 2:** Process of obtaining expressions for the sidelobes of the PACF.

	a_1_	a_2_	a_3_	a_4_			
m	−3	−2	−1	0	1	2	3
a_4_*	a_1_ × a_4_*	a_2_ × a_4_*	a_3_ × a_4_*	a_4_ × a_4_*	a_1_ × a_4_*	a_2_ × a_4_*	a_3_ × a_4_*
a_3_*	a_4_ × a_3_*	a_1_ × a_3_*	a_2_ × a_3_*	a_3_ × a_3_*	a_4_ × a_3_*	a_1_ × a_3_*	a_2_ × a_3_*
a_2_*	a_3_ × a_2_*	a_4_ × a_2_*	a_1_ × a_2_*	a_2_ × a_2_*	a_3_ × a_2_*	a_4_ × a_2_*	a_1_ × a_2_*
a_1_*	a_2_ × a_1_*	a_3_ × a_1_*	a_4_ × a_1_*	a_1_ × a_1_*	a_2_ × a_1_*	a_3_ × a_1_*	a_4_ × a_1_*
	a_1_ × a_4_* + a_4_ × a_3_* + a_3_ × a_2_* + a_2_ × a_1_*	a_2_ × a_4_* + a_1_ × a_3_* + a_4_ × a_2_* + a_3_* a_1_*	a_3_ × a_4_* + a_2_ × a_3_* + a_1_ × a_2_* + a_4_ × a_1_*	a_4_ × a_4_* + a_3_ × a_3_* + a_2_ × a_2_* + a_1_ × a_1_*	a_1_ × a_4_* + a_4_ × a_3_* + a_3_ × a_2_* + a_2_ × a_1_*	a_2_ × a_4_* + a_1_ × a_3_* + a_4_ × a_2_* + a_3_ × a_1_*	a_3_ × a_4_* + a_2_ × a_3_* + a_1_ × a_2_* + a_1_ × a_1_*

**Table 3:** M-sequences and the polynomials that generate them.

№	Length	Generating Polynomial	M-Sequence
1	7	*x*^3^ + *x*^2^ + 1	−1 −1 1 1 1 −1 1
2	7	*x*^3^ + *x* + 1	1 1 −1 −1 1 −1 1
3	15	*x*^4^ + *x* + 1	1 −1 1 −1 1 1 1 1 −1 −1 −1 1 −1 −1 1
4	15	*x*^4^ + *x*^3^ + 1	−1 −1 1 1 1 1 −1 1 −1 1 1 −1 −1 1 −1

**Table 4:** M-sequences with alphabet {1; −exp(φi)}.

№	Length	M-Sequence
1	7	−exp(φi) −exp(φi) 1 1 1 −exp(φi) 1
2	7	1 1 −exp(φi) −exp(φi) 1 −exp(φi) 1
3	15	1 −exp(φi) 1 −exp(φi) 1 1 1 1 −exp(φi) −exp(φi) −exp(φi) 1 −exp(φi) −exp(φi) 1
4	15	−exp(φi) −exp(φi) 1 1 1 1 −exp(φi) 1 −exp(φi) 1 1 −exp(φi) −exp(φi) 1 −exp(φi)

**Table 5:** M-sequences with alphabet {exp(φ_1_i); −exp(φ_2_i)}.

№	Length	M-Sequence
1	7	−exp(φ_2_*i*) −exp(φ_2_*i*) exp(φ_1_*i*) exp(φ_1_*i*) exp(φ_1_*i*) −exp(φ_2_*i*) exp(φ_1_*i*)
2	7	exp(φ_1_*i*) exp(φ_1_*i*) −exp(φ_2_*i*) −exp(φ_2_*i*) exp(φ_1_*i*) −exp(φ_2_*i*) exp(φ_1_*i*)
3	15	exp(φ_1_*i*) −exp(φ_2_*i*) exp(φ_1_*i*) −exp(φ_2_*i*) exp(φ_1_*i*) exp(φ_1_*i*) exp(φ_1_*i*) exp(φ_1_*i*) −exp(φ_2_*i*) −exp(φ_2_*i*) −exp(φ_2_*i*) exp(φ_1_*i*) −exp(φ_2_*i*) −exp(φ_2_*i*) exp(φ_1_*i*)
4	15	−exp(φ_2_*i*) −exp(φ_2_*i*) exp(φ_1_*i*) exp(φ_1_*i*) exp(φ_1_*i*) exp(φ_1_*i*) −exp(φ_2_*i*) exp(φ_1_*i*) −exp(φ_2_*i*) exp(φ_1_*i*) exp(φ_1_*i*) −exp(φ_2_*i*) −exp(φ_2_*i*) exp(φ_1_*i*) −exp(φ_2_*i*)

**Table 6:** M-sequences of length N = 7, 15, 31, 63, 127, 255, 511.

Length	Generating Polynomial	Initial Register
7	*x*^3^ + *x*^2^ + 1	[1 0 0]
	*x*^3^ + *x* + 1	[0 1 1]
15	*x*^4^ + *x* + 1	[0 1 0 1]
	*x*^4^ + *x*^3^ + 1	[1 1 0 0]
31	*x*^5^ + *x*^2^ + 1	[1 0 1 1 0]
	*x*^5^ + *x*^3^ + 1	[0 1 0 1 1]
	*x*^5^ + *x*^3^ + *x*^2^ + *x* + 1	[0 1 1 0 1]
	*x*^5^ + *x*^4^ + *x*^3^ + *x* + 1	[1 0 0 1 0]
	*x*^5^ + *x*^4^ + *x*^3^ + *x*^2^ + 1	[1 0 1 1 0]
	*x*^5^ + *x*^4^ + *x*^2^ + *x* + 1	[0 1 0 0 1]
63	*x*^6^ + *x* + 1	[1 0 1 0 1 1]
	*x*^6^ + *x*^4^ + *x*^3^ + *x* + 1	[0 1 0 0 1 1]
	*x*^6^ + *x*^5^ + 1	[1 0 0 1 1 1]
	*x*^6^ + *x*^5^ + *x*^2^ + *x* + 1	[1 0 1 1 0 0]
	*x*^6^ + *x*^5^ + *x*^3^ + *x*^2^ + 1	[0 1 0 0 0 1]
	*x*^6^ + *x*^5^ + *x*^4^ + *x* + 1	[1 0 1 0 0 1]
127	*x*^7^ + *x* + 1	[1 0 0 1 0 1 1]
	*x*^7^ + *x*^3^ + 1	[1 0 1 1 1 0 0]
	*x*^7^ + *x*^3^ + *x*^2^ + *x* + 1	[1 0 0 1 1 0 1]
	*x*^7^ + *x*^4^ + 1	[1 1 0 1 0 0 1]
	*x*^7^ + *x*^4^ + *x*^3^ + *x*^2^ + 1	[0 1 0 1 0 1 1]
	*x*^7^ + *x*^5^ + *x*^2^ + *x* + 1	[1 1 0 0 0 1 0]
	*x*^7^ + *x*^5^ + *x*^3^ + *x* + 1	[1 0 0 1 0 1 1]
	*x*^7^ + *x*^5^ + *x*^4^ + *x*^3^ + 1	[0 0 1 0 0 1 1]
	*x*^7^ + *x*^5^ + *x*^4^ + *x*^3^ + *x*^2^ + *x* + 1	[0 1 0 0 1 0 1]
	*x*^7^ + *x*^6^ + 1	[1 0 0 1 0 0 1]
	*x*^7^ + *x*^6^ + *x*^3^ + *x* + 1	[0 1 1 0 0 1 0]
	*x*^7^ + *x*^6^ + *x*^4^ + *x*^2^ + 1	[1 0 0 1 0 0 1]
	*x*^7^ + *x*^6^ + *x*^4^ + *x* + 1	[0 1 0 0 1 1 0]
	*x*^7^ + *x*^6^ + *x*^5^ + *x*^2^ + 1	[1 0 1 0 1 0 1]
	*x*^7^ + *x*^6^ + *x*^5^ + *x*^3^ + *x*^2^ + *x* + 1	[0 0 1 0 0 1 1]
	*x*^7^ + *x*^6^ + *x*^5^ + *x*^4^ + 1	[1 1 0 0 1 0 0]
	*x*^7^ + *x*^6^ + *x*^5^ + *x*^4^ + *x*^2^ + *x* + 1	[0 1 0 1 0 0 1]
	*x*^7^ + *x*^6^ + *x*^5^ + *x*^4^ + *x*^3^ + *x*^2^ + 1	[1 0 0 1 0 1 0]
255	*x*^8^ + *x*^4^ + *x*^3^ + *x*^2^ + 1	[1 0 1 1 0 1 0 1]
	*x*^8^ + *x*^5^ + *x*^3^ + *x* + 1	[1 0 1 0 1 0 0 1]
	*x*^8^ + *x*^5^ + *x*^3^ + *x*^2^ + 1	[0 1 0 0 1 1 0 1]
	*x*^8^ + *x*^6^ + *x*^3^ + *x*^2^ + 1	[1 0 1 1 0 0 1 0]
	*x*^8^ + *x*^6^ + *x*^5^ + *x* + 1	[1 1 0 1 0 0 1 1]
	*x*^8^ + *x*^6^ + *x*^5^ + *x*^2^ + 1	[0 1 0 0 1 0 1 1]
	*x*^8^ + *x*^6^ + *x*^5^ + *x*^3^ + 1	[1 1 0 0 0 1 1 0]
	*x*^8^ + *x*^6^ + *x*^5^ + *x*^4^ + 1	[0 1 0 1 1 0 0 1]
	*x*^8^ + *x*^6^ + *x*^4^ + *x*^3^ + *x*^2^ + *x* + 1	[1 0 1 1 0 1 0 1]
	*x*^8^ + *x*^6^ + *x*^5^ + *x* + 1	[0 1 0 1 0 0 1 1]
	*x*^8^ + *x*^7^ + *x*^2^ + *x* + 1	[1 1 0 0 1 1 0 1]
	*x*^8^ + *x*^7^ + *x*^3^ + *x*^2^ + 1	[1 1 0 1 1 0 1 0]
	*x*^8^ + *x*^7^ + *x*^5^ + *x*^3^ + 1	[1 1 1 0 0 1 1 0]
	*x*^8^ + *x*^7^ + *x*^6^ + *x*^5^ + *x*^2^ + *x* + 1	[0 0 1 0 1 1 0 1]
	*x*^8^ + *x*^7^ + *x*^6^ + *x*^5^ + *x*^4^ + *x*^2^ + 1	[1 0 1 1 0 1 1 0]
	*x*^8^ + *x*^7^ + *x*^6^ + *x* + 1	[0 1 0 0 1 0 1 1]
511	*x*^9^ + *x*^4^ + 1	[1 0 1 1 0 1 1 1 0]
	*x*^9^ + *x*^4^ + *x*^3^ + *x* + 1	[1 1 1 0 1 0 1 0 0]
	*x*^9^ + *x*^5^ + 1	[0 1 1 0 1 0 1 1 0]
	*x*^9^ + *x*^5^ + *x*^3^ + *x*^2^ + 1	[1 0 1 0 0 1 1 0 0]
	*x*^9^ + *x*^5^ + *x*^4^ + *x* + 1	[1 0 1 0 0 1 0 0 1]
	*x*^9^ + *x*^6^ + *x*^4^ + *x*^3^ + 1	[1 1 0 0 1 1 0 0 1]
	*x*^9^ + *x*^7^ + *x*^2^ + *x* + 1	[0 1 0 1 1 0 0 1 0]
	*x*^9^ + *x*^7^ + *x*^5^ + *x* + 1	[0 1 0 0 0 1 1 1 0]
	*x*^9^ + *x*^7^ + *x*^5^ + *x*^2^ + 1	[1 0 0 1 1 1 0 0 1]
	*x*^9^ + *x*^7^ + *x*^6^ + *x*^4^ + 1	[1 0 1 1 0 0 1 0 0]
	*x*^9^ + *x*^8^ + *x*^6^ + *x*^5^ + *x*^4^ + *x*^3^ + *x*^2^ + *x* + 1	[1 1 0 1 1 0 0 1 0]
	*x*^9^ + *x*^8^ + *x*^7^ + *x*^6^ + *x*^5^ + *x*^4^ + *x*^3^ + *x* + 1	[0 1 0 1 1 1 0 1 1]

**Table 7:** Comparison of SL of the NACF levels.

Length	Generating Polynomial	Average Level of NACF M-Sequences, dB					
		Aperiodic			Periodic		
		Traditional	Complex	Difference	Traditional	Complex	Difference
7	*x*^3^ + *x*^2^ + 1	−10.88	−13.89	3.01	−16.90	−112.04	95.14
	*x*^3^ + *x* + 1	−10.88	−16.89	6.01			
15	*x*^4^ + *x* + 1	−13.98	−14.63	0.65	−23.52	−93.29	69.77
	*x*^4^ + *x*^3^ + 1	−11.48	−12.87	1.39			
31	*x*^5^ + *x*^2^ + 1	−15.85	−17.77	1.92	−29.82	−97.72	67.90
	*x*^5^ + *x*^3^ + 1	−15.85	−16.82	0.97			
	*x*^5^ + *x*^3^ + *x*^2^ + *x* + 1	−15.85	−17.12	1.27			
	*x*^5^ + *x*^4^ + *x*^3^ + *x* + 1	−17.79	−18.46	0.67			
	*x*^5^ + *x*^4^ + *x*^3^ + *x*^2^ + 1	−15.85	−16.62	0.77			
	*x*^5^ + *x*^4^ + *x*^2^ + *x* + 1	−16.38	−17.12	0.74			
63	*x*^6^ + *x* + 1	−16.90	−17.63	0.73	−35.97	−95.65	69.68
	*x*^6^ + *x*^4^ + *x*^3^ + *x* + 1	−16.90	−17.67	0.77			
	*x*^6^ + *x*^5^ + 1	−16.90	−18.03	1.13			
	*x*^6^ + *x*^5^ + *x*^2^ + *x* + 1	−17.93	−18.23	0.30			
	*x*^6^ + *x*^5^ + *x*^3^ + *x*^2^ + 1	−16.90	−17.65	0.75			
	*x*^6^ + *x*^5^ + *x*^4^ + *x* + 1	−19.09	−20.30	1.21			
127	*x*^7^ + *x* + 1	−19.72	−19.74	0.02	−42.05	−100.92	58.74
	*x*^7^ + *x*^3^ + 1	−20.49	−21.18	0.69			
	*x*^7^ + *x*^3^ + *x*^2^ + *x* + 1	−20.49	−20.68	0.19			
	*x*^7^ + *x*^4^ + 1	−21.25	−21.85	0.60			
	*x*^7^ + *x*^4^ + *x*^3^ + *x*^2^ + 1	−21.25	−21.57	0.32			
	*x*^7^ + *x*^5^ + *x*^2^ + *x* + 1	−21.25	−21.72	0.47			
	*x*^7^ + *x*^5^ + *x*^3^ + *x* + 1	−21.25	−21.46	1.21			
	*x*^7^ + *x*^5^ + *x*^4^ + *x*^3^ + 1	−20.49	−21.09	0.6			
	*x*^7^ + *x*^5^ + *x*^4^ + *x*^3^ + *x*^2^ + *x* + 1	−19.80	−20.07	0.27			
	*x*^7^ + *x*^6^ + 1	−21.25	−21.83	0.58			
	*x*^7^ + *x*^6^ + *x*^3^ + *x* + 1	−21.25	−21.61	0.36			
	*x*^7^ + *x*^6^ + *x*^4^ + *x*^2^ + 1	−19.15	−19.62	0.47			
	*x*^7^ + *x*^6^ + *x*^4^ + *x* + 1	−22.08	−22.47	0.39			
	*x*^7^ + *x*^6^ + *x*^5^ + *x*^2^ + 1	−20.49	−21.11	0.62			
	*x*^7^ + *x*^6^ + *x*^5^ + *x*^3^ + *x*^2^ + *x* + 1	−22.08	−22.26	0.18			
	*x*^7^ + *x*^6^ + *x*^5^ + *x*^4^ + 1	−22.08	−22.32	0.24			
	*x*^7^ + *x*^6^ + *x*^5^ + *x*^4^ + *x*^2^ + *x* + 1	−21.25	−21.44	0.19			
	*x*^7^ + *x*^6^ + *x*^5^ + *x*^4^ + *x*^3^ + *x*^2^ + 1	−21.25	−21.62	0.37			
255	*x*^8^ + *x*^4^ + *x*^3^ + *x*^2^ + 1	−24.61	−25.03	0.42	−48.18	−102.73	54.55
	*x*^8^ + *x*^5^ + *x*^3^ + *x* + 1	−23.52	−23.73	0.21			
	*x*^8^ + *x*^5^ + *x*^3^ + *x*^2^ + 1	−23.03	−23.31	0.28			
	*x*^8^ + *x*^6^ + *x*^3^ + *x*^2^ + 1	−22.11	−22.37	0.26			
	*x*^8^ + *x*^6^ + *x*^5^ + *x* + 1	−24.05	−24.41	0.36			
	*x*^8^ + *x*^6^ + *x*^5^ + *x*^2^ + 1	−23.52	−23.82	0.30			
	*x*^8^ + *x*^6^ + *x*^5^ + *x*^3^ + 1	−23.03	−23.33	0.30			
	*x*^8^ + *x*^6^ + *x*^5^ + *x*^4^ + 1	−24.61	−24.84	0.23			
	*x*^8^ + *x*^6^ + *x*^4^ + *x*^3^ + *x*^2^ + *x* + 1	−23.52	−23.86	0.34			
	*x*^8^ + *x*^6^ + *x*^5^ + *x* + 1	−24.05	−24.28	0.23			
	*x*^8^ + *x*^7^ + *x*^2^ + *x* + 1	−24.05	−24.35	0.30			
	*x*^8^ + *x*^7^ + *x*^3^ + *x*^2^ + 1	−23.03	−23.41	0.38			
	*x*^8^ + *x*^7^ + *x*^5^ + *x*^3^ + 1	−23.52	−23.75	0.23			
	*x*^8^ + *x*^7^ + *x*^6^ + *x*^5^ + *x*^2^ + *x* + 1	−23.52	−23.69	0.17			
	*x*^8^ + *x*^7^ + *x*^6^ + *x*^5^ + *x*^4^ + *x*^2^ + 1	−24.05	−24.42	0.63			
	*x*^8^ + *x*^7^ + *x*^6^ + *x* + 1	−23.52	−23.79	0.27			
511	*x*^9^ + *x*^4^ + 1	−27.32	−27.62	0.30	−53.98	−110.17	56.19
	*x*^9^ + *x*^4^ + *x*^3^ + *x* + 1	−27.32	−27.43	0.11			
	*x*^9^ + *x*^5^ + 1	−27.32	−27.19	0.13			
	*x*^9^ + *x*^5^ + *x*^3^ + *x*^2^ + 1	−24.92	−25.18	0.26			
	*x*^9^ + *x*^5^ + *x*^4^ + *x* + 1	−26.93	−27.05	0.12			
	*x*^9^ + *x*^6^ + *x*^4^ + *x*^3^ + 1	−25.87	−26.06	0.19			
	*x*^9^ + *x*^7^ + *x*^2^ + *x* + 1	−26.21	−26.40	0.19			
	*x*^9^ + *x*^7^ + *x*^5^ + *x* + 1	−24.92	−25.03	0.11			
	*x*^9^ + *x*^7^ + *x*^5^ + *x*^2^ + 1	−26.93	−27.14	0.21			
	*x*^9^ + *x*^7^ + *x*^6^ + *x*^4^ + 1	−26.93	−27.26	0.33			
	*x*^9^ + *x*^8^ + *x*^6^ + *x*^5^ + *x*^4^ + *x*^3^ + *x*^2^ +*x* + 1	−26.56	−26.70	0.14			
	*x*^9^ + *x*^8^ + *x*^7^ + *x*^6^ + *x*^5^ + *x*^4^ + *x*^3^ + *x* + 1	−26.93	−27.05	0.12			

**Table 8:** Numerical comparison of the SL levels of compressed signals modulated in phase based on traditional and new complex M-sequences.

Length	Generating Polynomial	Average Level of NACF M-Sequences, dB					
		Aperiodic			Periodic		
		Traditional	Complex	Difference	Traditional	Complex	Difference
7	*x*^3^ + *x*^2^ + 1	−10.88	−14.25	3.37	−16.91	−112.01	95.10
	*x*^3^ + *x* + 1	−10.88	−16.90	6.02			
15	*x*^4^ + *x* + 1	−13.98	−15.06	1.08	−23.51	−93.27	69.76
	*x*^4^ + *x*^3^ + 1	−11.48	−12.94	1.48			
31	*x*^5^ + *x*^2^ + 1	−15.85	−16.83	0.98	−29.82	−97.70	67.88
	*x*^5^ + *x*^3^ + 1	−15.85	−17.79	1.94			
	*x*^5^ + *x* ^3^ + *x* ^2^ + *x* + 1	−15.85	−17.11	1.26			
	*x*^5^ + *x*^4^ + *x*^3^ + *x* + 1	−17.79	−18.46	0.67			
	*x*^5^ + *x*^4^ + *x*^3^ + *x*^2^ + 1	−15.85	−16.60	0.75			
	*x*^5^ + *x*^4^ + *x*^2^ + *x* + 1	−16.38	−17.11	0.73			
63	*x*^6^ + *x* + 1	−16.90	−17.61	0.71	−35.97	−95.64	59.67
	*x*^6^ + *x*^4^ + *x*^3^ + *x* + 1	−16.90	−17.67	0.77			
	*x*^6^ + *x*^5^ + 1	−16.90	−18.03	1.13			
	*x*^6^ + *x*^5^ + *x*^2^ + *x* + 1	−17.93	−18.25	0.32			
	*x*^6^ + *x*^5^ + *x*^3^ + *x*^2^ + 1	−16.90	−17.65	0.75			
	*x*^6^ + *x*^5^ + *x*^4^ + *x* + 1	−19.09	−20.31	1.22			
127	*x*^7^ + *x* + 1	−19.72	−19.76	0.04	−42.06	−100.93	58.87
	*x*^7^ + *x*^3^ + 1	−20.49	−21.19	0.70			
	*x*^7^ + *x*^3^ + *x*^2^ + *x* + 1	−20.49	−20.68	0.19			
	*x*^7^ + *x*^4^ + 1	−21.25	−21.84	0.59			
	*x*^7^ + *x*^4^ + *x*^3^ + *x*^2^ + 1	−21.25	−21.58	0.33			
	*x*^7^ + *x*^5^ + *x*^2^ + *x* + 1	−21.25	−21.72	0.47			
	*x*^7^ + *x*^5^ + *x*^3^ + *x* + 1	−21.25	−21.47	0.22			
	*x*^7^ + *x*^5^ + *x*^4^ + *x*^3^ + 1	−20.49	−21.08	0.59			
	*x*^7^ + *x*^5^ + *x*^4^ + *x*^3^ + *x*^2^ + *x* + 1	−19.80	−20.08	0.28			
	*x*^7^ + *x*^6^ + 1	−21.25	−21.83	0.58			
	*x*^7^ + *x*^6^ + *x*^3^ + *x* + 1	−21.25	−21.60	0.35			
	*x*^7^ + *x*^6^ + *x*^4^ + *x*^2^ + 1	−19.15	−19.60	0.45			
	*x*^7^ + *x*^6^ + *x*^4^ + *x* + 1	−22.08	−22.47	0.39			
	*x*^7^ + *x*^6^ + *x*^5^ + *x*^2^ + 1	−20.49	−21.11	0.62			
	*x*^7^ + *x*^6^ + *x*^5^ + *x*^3^ + *x*^2^ + *x* + 1	−22.08	−22.26	0.18			
	*x*^7^ + *x*^6^ + *x*^5^ + *x*^4^ + 1	−22.08	−22.30	0.22			
	*x*^7^ + *x*^6^ + *x*^5^ + *x*^4^ + *x*^2^ + *x* + 1	−21.25	−21.44	1.19			
	*x*^7^ + *x*^6^ + *x*^5^ + *x*^4^ + *x*^3^ + *x*^2^ + 1	−21.25	−21.63	0.38			
255	*x*^8^ + *x*^4^ + *x*^3^ + *x*^2^ + 1	−24.61	−25.03	0.42	−48.18	−102.72	54.54
	*x*^8^ + *x*^5^ + *x*^3^ + *x* + 1	−23.52	−23.75	0.23			
	*x*^8^ + *x*^5^ + *x*^3^ + *x*^2^ + 1	−23.03	−23.31	0.28			
	*x*^8^ + *x*^6^ + *x*^3^ + *x*^2^ + 1	−22.11	−22.38	0.27			
	*x*^8^ + *x*^6^ + *x*^5^ + *x* + 1	−24.05	−24.41	0.36			
	*x*^8^ + *x*^6^ + *x*^5^ + *x*^2^ + 1	−23.52	−23.81	0.29			
	*x*^8^ + *x*^6^ + *x*^5^ + *x*^3^ + 1	−23.03	−23.33	0.30			
	*x*^8^ + *x*^6^ + *x*^5^ + *x*^4^ + 1	−24.61	−24.85	0.76			
	*x*^8^ + *x*^6^ + *x*^4^ + *x*^3^ + *x*^2^ + *x* + 1	−23.52	−23.86	0.34			
	*x*^8^ + *x*^6^ + *x*^5^ + *x* + 1	−24.05	−24.27	0.22			
	*x*^8^ + *x*^7^ + *x*^2^ + *x* + 1	−24.05	−24.34	0.27			
	*x*^8^ + *x*^7^ + *x*^3^ + *x*^2^ + 1	−23.03	−23.40	0.37			
	*x*^8^ + *x*^7^ + *x*^5^ + *x*^3^ + 1	−23.52	−23.74	0.22			
	*x*^8^ + *x*^7^ + *x*^6^ + *x*^5^ + *x*^2^ + *x* + 1	−23.52	−23.69	0.17			
	*x*^8^ + *x*^7^ + *x*^6^ + *x*^5^ + *x*^4^ + *x*^2^ + 1	−24.05	−24.41	0.36			
	*x*^8^ + *x*^7^ + *x*^6^ + *x* + 1	−23.52	−23.78	0.26			
511	*x*^9^ + *x*^4^ + 1	−27.32	−27.61	0.29	−53.99	−110.18	56.19
	*x*^9^ + *x*^4^ + *x*^3^ + *x* + 1	−27.32	−27.44	0.12			
	*x*^9^ + *x*^5^ + 1	−27.32	−27.19	0.13			
	*x*^9^ + *x*^5^ + *x*^3^ + *x*^2^ + 1	−24.92	−25.17	0.25			
	*x*^9^ + *x*^5^ + *x*^4^ + *x* + 1	−26.93	−27.04	0.11			
	*x*^9^ + *x*^6^ + *x*^4^ + *x*^3^ + 1	−25.87	−26.07	0.20			
	*x*^9^ + *x*^7^ + *x*^2^ + *x* + 1	−26.21	−26.42	0.21			
	*x*^9^ + *x*^7^ + *x*^5^ + *x* + 1	−24.92	−25.02	0.10			
	*x*^9^ + *x*^7^ + *x*^5^ + *x*^2^ + 1	−26.93	−27.12	0.19			
	*x*^9^ + *x*^7^ + *x*^6^ + *x*^4^ + 1	−26.93	−27.25	0.32			
	*x*^9^ + *x*^8^ + *x*^6^ + *x*^5^ + *x*^4^ + *x*^3^ + *x*^2^ + *x* + 1	−26.56	−26.70	0.14			
	*x*^9^ + *x*^8^ + *x*^7^ + *x*^6^ + *x*^5^ + *x*^4^ + *x*^3^ + *x* + 1	−26.93	−27.05	0.12			

## Data Availability

All data supporting the reported results are public.

## Abbreviations

The following abbreviations are used in this manuscript:

ACF	Autocorrelation function
AACF	Aperiodic autocorrelation function
NACF	Normalized autocorrelation function
CCF	Cross-correlation function
PACF	Periodic autocorrelation function
PM	Phase modulation
SL	Side lobe
CORS	Onboard radar systems
